# A Defined Antigen Skin Test That Enables Implementation of BCG Vaccination for Control of Bovine Tuberculosis: Proof of Concept

**DOI:** 10.3389/fvets.2020.00391

**Published:** 2020-07-24

**Authors:** Sreenidhi Srinivasan, Saraswathi Subramanian, Sai Shankar Balakrishnan, Kathiravan Ramaiyan Selvaraju, Vandana Manomohan, Suganya Selladurai, Monika Jothivelu, Srinivasan Kandasamy, Dhinakar Raj Gopal, Kumanan Kathaperumal, Andrew J. K. Conlan, Maroudam Veerasami, Douwe Bakker, Martin Vordermeier, Vivek Kapur

**Affiliations:** ^1^Department of Animal Science, The Pennsylvania State University, University Park, PA, United States; ^2^The Huck Institutes of the Life Sciences, The Pennsylvania State University, University Park, PA, United States; ^3^Translational Research Platform for Veterinary Biologicals, Centre for Animal Health Studies, Tamil Nadu Veterinary and Animal Sciences University, Chennai, India; ^4^Disease Dynamics Unit, Department of Veterinary Medicine, University of Cambridge, Cambridge, United Kingdom; ^5^Cisgen Biotech Discoveries Private Limited, Chennai, India; ^6^Independent Researcher and Technical Consultant, Lelystad, Netherlands; ^7^Animal and Plant Health Agency, Weybridge, United Kingdom; ^8^Centre for Bovine Tuberculosis, Institute for Biological, Environmental and Rural Sciences, University of Aberystwyth, Aberystwyth, United Kingdom

**Keywords:** bovine tuberculosis, BCG, specificity, DIVA, DST

## Abstract

In most low- and middle-income countries (LMICs), bovine tuberculosis (bTB) remains endemic due to the absence of control programs. This is because successful bTB control and eradication programs have relied on test-and-slaughter strategies that are socioeconomically unfeasible in LMICs. While Bacillus Calmette–Guérin (BCG) vaccine-induced protection for cattle has long been documented in experimental and field trials, its use in control programs has been precluded by the inability to differentiate BCG-vaccinated from naturally infected animals using the OIE-prescribed purified protein derivative (PPD)-based tuberculin skin tests. In the current study, the diagnostic specificity and capability for differentiating infected from vaccinated animals (DIVA) of a novel defined antigen skin test (DST) in BCG-vaccinated (*Bos taurus* ssp. *taurus x B. t*. ssp. *indicus*) calves were compared with the performance of traditional PPD-tuberculin in both the skin test and *in vitro* interferon-gamma release assay (IGRA). The IFN-γ production from whole blood cells stimulated with both PPDs increased significantly from the 0 week baseline levels, while DST induced no measurable IFN-γ production in BCG-vaccinated calves. None of the 15 BCG-vaccinated calves were reactive with the DST skin test (100% specificity; one-tailed lower 95% CI: 82). In contrast, 10 of 15 BCG-vaccinated calves were classified as reactors with the PPD-based single intradermal test (SIT) (specificity in vaccinated animals = 33%; 95% CI: 12, 62). Taken together, the results provide strong evidence that the DST is highly specific and enables DIVA capability in both skin and IGRA assay format, thereby enabling the implementation of BCG vaccine-based bTB control, particularly in settings where test and slaughter remain unfeasible.

## Introduction

Bovine tuberculosis (bTB) is a chronic, granulomatous, inflammatory disease that is predominantly caused by members of the *Mycobacterium tuberculosis* complex (MTBC) in cattle. The disease poses a significant threat to public health and limits livestock productivity, resulting in an estimated economic burden of $3 billion globally, annually (1). While bTB is well-controlled in most high-income countries, it remains endemic in low- and middle-income countries (LMICs) where national control programs have not yet been implemented due to socio-economic reasons (2–4). Further, the predicted intensification of dairy and cattle farming will likely increase the associated zoonotic risks, and the economic burden of the disease in the coming years (5–7). Thus, there is a well-recognized urgent need for alternate intervention programs such as vaccination to be implemented in these geographies.

Bacillus Calmette–Guérin (BCG) is a live attenuated strain of *M. bovis* that was initially isolated from the udder of a tuberculous cow (8, 9). This *M. bovis* strain was serially passaged for a period of 13 years (1908–1921) at the Pasteur Institute and since has been used as a vaccine against TB in humans. First evaluation of BCG against bTB in cattle occurred 10 years prior, in 1912, and following promising initial reports, the vaccine has been extensively evaluated in both experimental and field trials. Encouragingly, numerous reports have demonstrated BCG-induced protection in field trials conducted in many different countries and settings (10). However, despite this promise, BCG has previously not been considered for widespread use as a livestock vaccine against bTB, primarily due to its cross-reactivity with the OIE-recommended purified protein derivatives (PPDs)-tuberculin based skin tests, resulting in an inability to differentiate naturally *M. tuberculosis* complex infected from BCG-vaccinated animals.

Currently, diagnosis of bTB in cattle is primarily based on the detection of T cell-mediated immune response. This is routinely performed using two diagnostic tests. The single intradermal test (SIT) is the international standard and is based on the use of PPD bovine (PPD-B)-tuberculin that represents culture media sterile filtrates containing a large number of antigens from a defined *M. bovis* strain, including those antigens that are broadly cross-reactive with the BCG vaccine (11). Upon injection in an infected animal, this elicits delayed-type hypersensitivity (DTH) reactions with an animal considered reactor if there is ≥4 mm increase in skin thickness at 72 h post-injection. In regions where exposure to environmental mycobacteria is an important confounding factor in the diagnosis of bTB, the comparative cervical tuberculin test (CCT) involving injection of both bovine and avian PPDs (extracts from a *M. avium* subsp. *avium* strain) is used to improve specificity, although this is known to compromise sensitivity (12). Also, an IFN-γ release assay (IGRA) is available as an ancillary test for improved detection of bTB-infected animals and is based on the release of IFN-γ by sensitized lymphocytes when re-exposed to MTBC antigens *in vitro* (13).

Efforts in recent years have been focused on developing BCG-compatible diagnostic tests with the ability to differentiate infected from vaccinated animals (DIVA). Genomic and transcriptomic analyses have identified ESAT-6, CFP10, and Rv3615c as promising antigens for differential diagnosis of bTB (14, 15). Recently, a novel defined antigen skin test (DST) consisting of peptides representing these three antigens has shown particular promise as a specific and sensitive test for the detection of bTB-infected animals in both experimental and field settings (16). While the specificity of this assay as a skin test in naïve bTB-free control animals was also previously determined, the specificity in BCG-vaccinated animals in field settings is unknown. In the context of a BCG-based national control program, the specificity of DST in correctly identifying disease-free vaccinates as uninfected is crucial.

Hence, the current investigation was designed to assess the utility of DST as a DIVA skin test in the face of BCG vaccination. The results show that DST possesses high specificity both as a skin test reagent and in *in vitro* assays in the face of BCG vaccination, suggesting that application of DST provides an opportunity to consider BCG vaccine-based bTB control programs in endemic settings, especially where test and slaughter are unfeasible.

## Materials and Methods

### Antigens and Peptides

Bovine tuberculin (PPD-B) and avian tuberculin (PPD-A) were purchased from Prionics, Thermo Fisher, Schlieren, Switzerland. The defined antigen skin test (DST) is a peptide-based cocktail representing three antigens from *M. bovis* viz, ESAT-6 (Mb3905, equivalent to Rv3875 in *M. tuberculosis* with 100% identity), CFP-10 (Mb3904, equivalent to Rv3874 in *M. tuberculosis* with 100% identity), and Rv3615c (equivalent to Mb3645c in *M. bovis* with 100% identity). A total of 13 peptides representing these three antigens were commercially synthesized at >98% purity by GenScript USA, Inc. and USV Private Limited, India (Supplementary Table 1 for peptide sequences). The *in vivo* safety of DST was confirmed in *B. taurus* ssp. *indicus* under Good Laboratory Practice (GLP) conditions in India with repeat and overdosing experiments (unpublished).

### Animals

**Figure 1:**
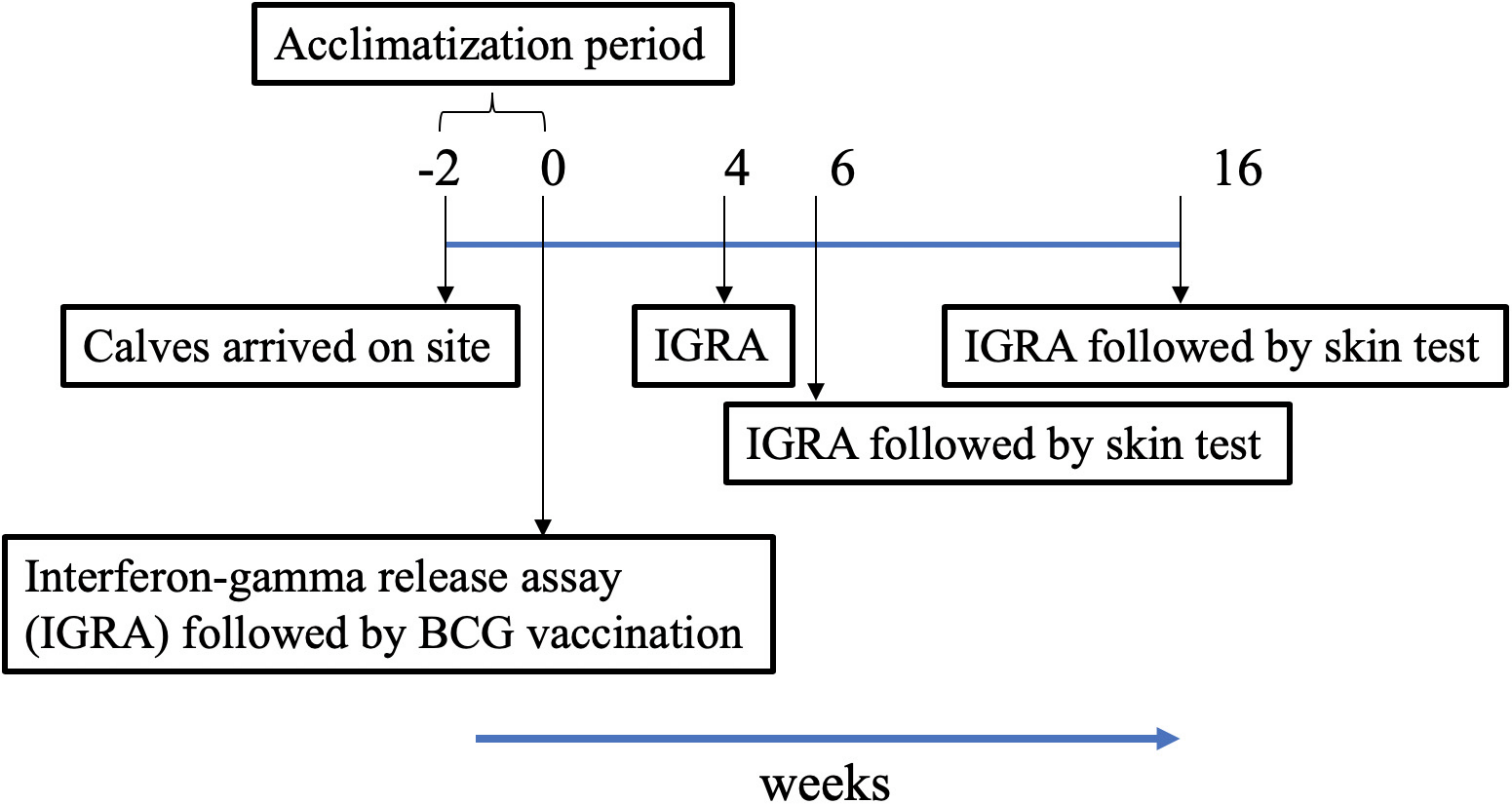
Timeline chart. Following arrival of calves on site, they were acclimatized for a period of 2 weeks. Vaccination of calves at week 0 denotes start of trial. Blood was collected for Interferon-gamma release assay (IGRA) just prior to injection of BCG, and at weeks 4, 6, and 16 post-vaccination. Skin tests were conducted at weeks 6 and 16.

The trial was conducted in India under field (normal animal husbandry) conditions. In order to assess the skin test performance of DST, 3- to 6-month-old crossbred calves (*B. taurus* ssp. *taurus* X *B. taurus* ssp. *indicus*) were recruited from bTB-free farms near Chennai, India. The population of crossbred cattle in India increased by 26.9% in 2019 as compared to the previous census (17). They are preferred in intensive farming due to their ability to produce high milk yields. Following recruitment, calves were housed in facilities at the Tamil Nadu Veterinary and Animal Sciences University (TANUVAS), and screened for helminths and dewormed during the acclimatization period of 2 weeks. During the trial period, calves were fed with milk initially and then with concentrate feed (a balanced mix of grains, brans, minerals, and vitamins prepared by the Central Feed Technology Unit at TANUVAS, and fed as a supplement to grasses and silage), green fodder, and water *ad libitum*. All cattle experiments were approved by the Institutional Animal Ethics Committee (IAEC) at TANUVAS and Committee for the Purpose of Control and Supervision of Experimental Animals (CPCSEA; F. No. 25/31/2017-CPCSEA). The trial timeline is shown in Figure 1.

### BCG Immunization

BCG Danish 1331 is among the most widely used TB vaccine strain for humans and was also used in the current trial. Freeze-dried preparations of BCG Danish strain were obtained from Green Signal Bio Pharma Pvt. Ltd., India. These were reconstituted as per-manufacturer instructions in 0.9% NaCl and plated in duplicates on modified 7H11 agar plates in order to determine the CFU of BCG (18). The plates were taped with parafilm and incubated in sealed bags for 28 days at 37C before counting the number of colonies on each plate. Calves were randomly assigned to two groups of 15 each. The vaccinates were subcutaneously administered a single dose (1–4 × 10^6^ CFU) of BCG Danish delivered in 0.5 ml, at ~3 to 6 months of age. This dose was decided based on previous studies that have shown relatively low doses of 10^4^-10^6^ CFU administered subcutaneously to be efficacious in inducing protective immunity (1).

### Intradermal Skin Test Procedures

PPD-tuberculins (PPD-B at 30,000 IU/ml and PPD-A at 25,000 IU/ml) were injected in a 0.1-ml volume as recommended by the manufacturer and the OIE. The DST injection is composed of 10 μg of each of the 13 peptides (0.1-ml final volume). The dose was based on prior dose titration experiments performed in cross-bred calves (*B. taurus* X *B. taurus* ssp. *indicus*) (19). The same operator measured skin thickness before injection and at 72 h post injection, and readings were recorded in millimeters as per OIE-prescribed guidelines (20).

### Interferon Gamma Enzyme-Linked Immunosorbent Assay (IFN-γ ELISA)

Bovine tuberculin (PPD-B) and avian tuberculin (PPD-A) antigens were purchased from Thermo Fisher Scientific. The BOVIGAM^TM^ kit was used for the IFN-γ ELISA. The tuberculin antigens (purchased from Thermo Fisher), PPD-B and PPD-A, were used for *in vitro* stimulation of whole blood at a final concentration of 300 and 250 IU/ml, respectively, as per the kit instructions (antigens provided with the BOVIGAM^TM^ kit were not used in the assays). The DST was used at 10 μg/ml in *in vitro* assays. Blood was collected for IGRA just prior to BCG vaccination at week 0, and just prior to skin test at weeks 4, 6, and 16. Whole blood was stimulated overnight (37C, 5% CO_2_) with antigens (PPDs and DST) *in vitro*. BOVIGAM^TM^ enzyme-linked immunosorbent assay-based kits (Thermo Fisher Scientific, USA) were used to determine IFN-γ concentrations in whole blood culture supernatants. Results for antigen-stimulated cultures were expressed as background-corrected optical density at 450 nm (i.e., ΔOD_450_).

### Statistical Analyses

Per the OIE guidelines for validation of diagnostic assays, this sample size of 15 animals per group gives 95% power to estimate a target specificity at least as good as CCT of ≥99.97% (with 5% precision) (20). Confidence intervals (CIs) for the specificity estimates in vaccinated animals for DST and PPD were calculated using the Clopper-Pearson method. For DST, with technical specificity of 100% (no false positives within sample), we calculated a one-sided CI (lower 95% CI: 82). Standard two-sided CIs were calculated for PPD using the same method with a point estimate of 33% (95% CI: 12, 62). All statistical analyses were performed using Prism 8 (GraphPad Software, La Jolla, CA). The Wilcoxon matched-pairs signed rank test (pairwise difference, single-tailed and *P* = 0.05) was used for comparing delta IFN-γ OD response between pre- and post-vaccination time points. The IGRAs were performed at four time points during the duration of the trial, the area under the curves (21) were determined and the statistical difference was compared using the Friedman (non-parametric) test. For skin test, statistical difference between responses induced by the various antigens was determined using the Friedman (non-parametric) test.

## Results

### DST Elicits Highly Specific IFN-γ Response in BCG Vaccinated Calves as Measured *in vitro*

**Figure 2:**
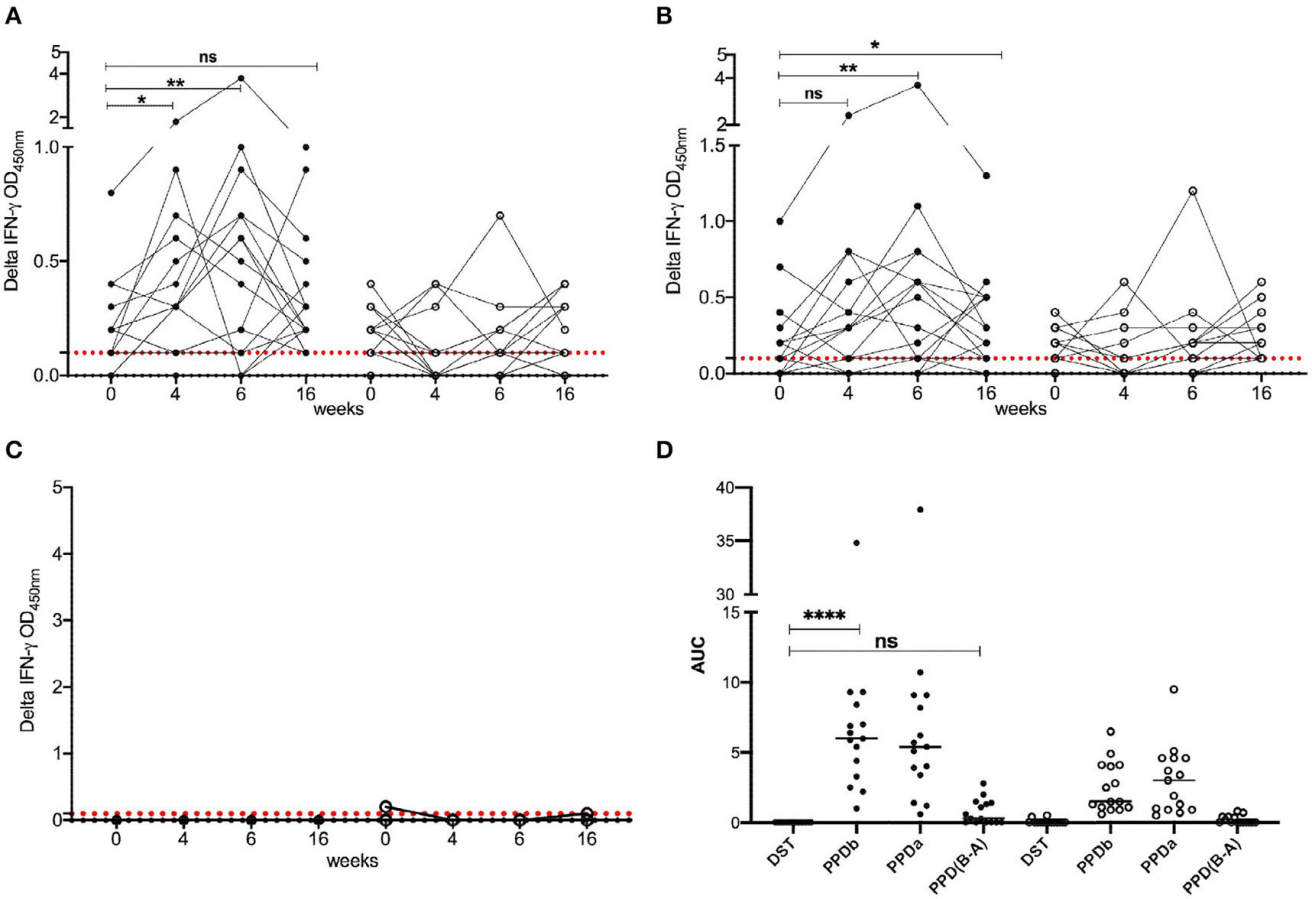
IFN-γ responses following BCG vaccination. Responses of BCG vaccinates (closed circle, *n* = 15) and controls (open circle, *n* = 15) to **(A)** PPD-B, **(B)** PPD-A, and **(C)** DST. The background-corrected (delta) optical density (OD) values are plotted. Time is shown as weeks post-vaccination in the *x*-axis. Calves were vaccinated with BCG at week 0. Blood was collected for IGRA just prior to injection of BCG, and at weeks 4, 6, and 16 post-vaccination. Skin test using PPDs and DST was conducted at weeks 6 and 16. The pairwise difference between pre- and post-vaccination time points was determined using the Wilcoxon matched pairs signed rank test (**P < 0.01; *P < 0.05). The dotted red line represents the IGRA cutoff of 0.1. **(D)** The bottom right panel provides the area under the curves (21), where the horizontal line provides the median, and the statistical difference between the responses was determined using Friedman (non-parametric) test (****P < 0.0001).

To determine the diagnostic performance of DST in *in vitro* assays, measurement of antigen-induced IFN-γ responses was performed using whole blood collected at weeks 0, 4, 6, and 16 post-vaccination from all BCG vaccinates and controls. Responses to avian and bovine PPD (PPD-A and PPD-B) were measured to determine vaccine-induced IFN-γ response Supplementary Table 1). At week 0, we detected low PPD-B and PPD-A responses in most animals in both groups most likely caused by cross-reaction with antigens from environmental mycobacteria, although 3/30 would be classified as IGRA-positive based on a PPD-B minus PPD-A > 0.1 OD value interpretation of the test (Supplementary Table 1). Given that the specificity of using tuberculin as stimulating antigen in IGRA is expected to be around 95%, this is not an unexpected test outcome, also given the fact that most animals at this time point were younger than 6 months, the minimum age cut-off for IGRA to avoid increased false-positive rates due to natural killer (NK) cell activity (22). The IFN-γ production from whole blood cells stimulated with both PPD-A and B increased significantly from the 0 week baseline levels in BCG-vaccinated calves at each of weeks 4, 6, and 16 (Table 1 and Figure 1). In contrast, no such increase from baseline levels was noted in the unvaccinated groups. While the observed group mean of IFN-γ responses to PPD-A and B appeared to peak at week 6 (at 0.63 ± 0.24 and 0.65 ± 0.24, respectively), there were no statistically significant differences among any of the post-vaccination time points (4, 6, or 16 weeks) (Table 1 and Figure 2). The results were also expressed as area under the curves to describe IGRA kinetic responses (AUC; Figure 2D). Also of note, the results showed no differences in the IFN-γ responses of whole blood from BCG-vaccinated animals stimulated with either of the PPDs, suggesting that BCG stimulates immune responses that are equally cross-reactive to antigens contained in PPD-B and PPD-A (Table 1). As expected, there were no statistical differences in the IFN-γ stimulation responses among any of the unvaccinated control calves to either of the PPDs at any time point (Table 1 and Figure 2).

**Table 1:** Summary of means of the IFN-γ responses stimulated by PPD-A, PPD-B, and DST across all time points studied, in all calves tested.

**Group**	**Antigen**	**Week**			
		**0**	**4**	**6**	**16**
BCG	PPD-B	0.21 (0.05)	0.45 (0.12)	0.65 (0.24)	0.37 (0.07)
	PPD-A	0.22 (0.07)	0.44 (0.16)	0.63 (0.24)	0.38 (0.08)
	DST	0 (0)	0 (0)	0 (0)	0 (0)
Control	PPD-B	0.19 (0.03)	0.10 (0.04)	0.15 (0.05)	0.19 (0.04)
	PPD-A	0.19 (0.03)	0.12 (0.05)	0.20 (0.08)	0.22 (0.04)
	DST	0.01 (0.01)	0 (0)	0 (0)	0.01 (0.01)

*The standard error of the mean is shown in parenthesis. Calves were vaccinated with BCG at week 0. Blood was collected for IGRA just prior to injection of BCG, and at weeks 4, 6, and 16 post-vaccination. Skin test using PPDs and DST was conducted at weeks 6 and 16*.

In stark contrast with the results of the PPDs, whole blood stimulated with DST did not result in any appreciable IFN-γ responses in either BCG vaccinated or unvaccinated control calves at any of the observed time points, suggesting that the DST is highly specific and exhibits no cross-reactivity in BCG vaccinated calves (Table 1 and Figure 2).

### DST Provides DIVA Capability in BCG Vaccinated Calves

**Figure 3:**
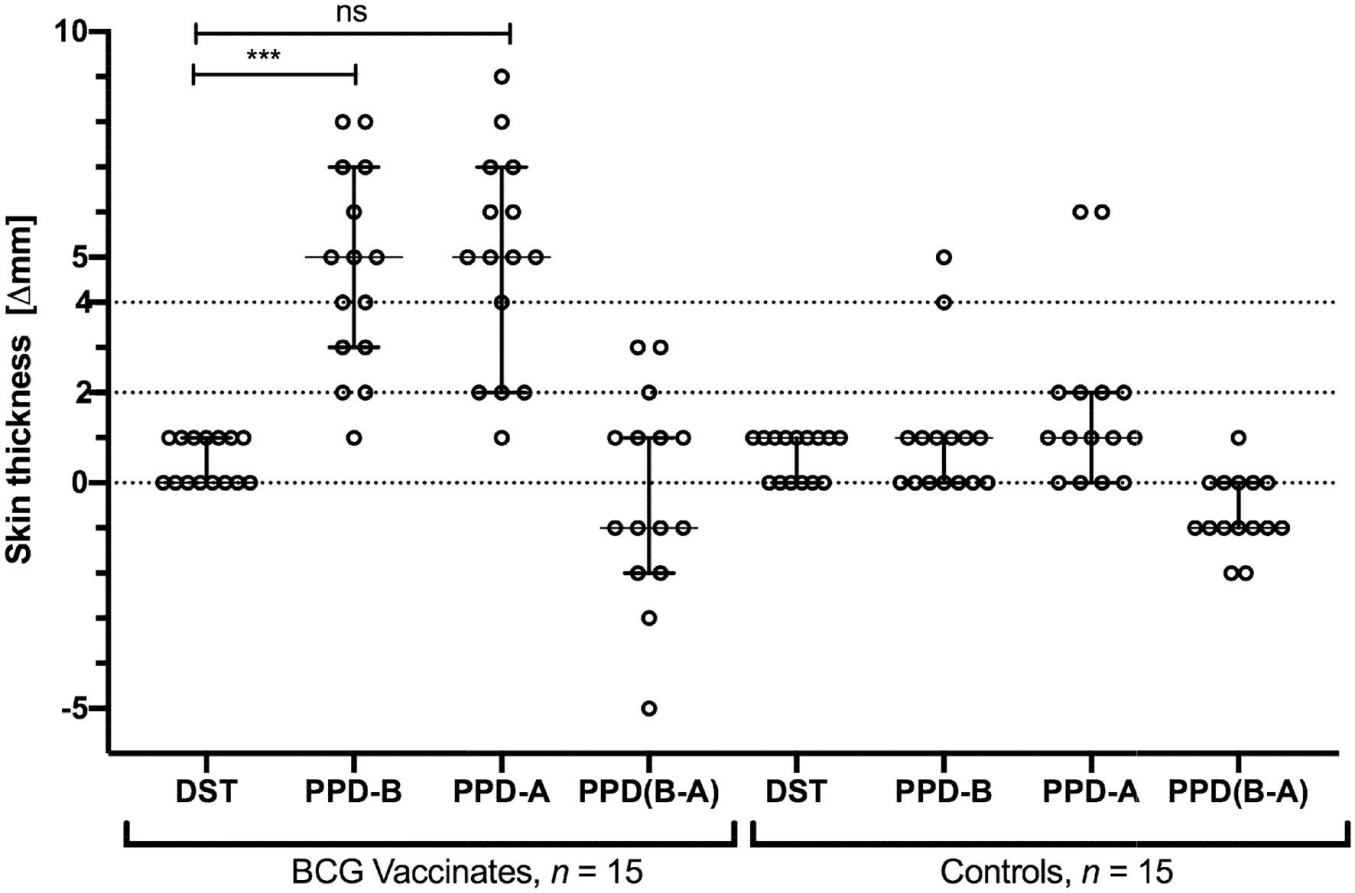
Skin test responses induced by DST, PPD-B, and PPD-A were measured 72 h post-injection in calves vaccinated with BCG (*n* = 15) and naïve controls (*n* = 15). Results are expressed as the difference in skin thickness (in millimeters) between pre- and post-skin test readings, with the horizontal line providing the median [±95% confidence interval (CI)]. The statistical difference between the responses was determined using Friedman test (***P < 0.001). The dotted horizontal lines at 2 and 4 mm are the cutoffs used for DST, and PPD-B and PPD (B-A), respectively. The two control animals that are SIT positive are the same two that show > 4 mm PPD-A responses.

We next tested specificity of DST in skin tests in BCG-vaccinated calves. Skin test responses to DST, PPD-A, and PPD-B were recorded 6 weeks post-BCG vaccination, the time point with the highest cell-mediated responses (Figure 2). The DST cocktail induced minimal, if any, increase in skin thickness in both BCG vaccinates and control calves 72 h post-injection (Figure 3). In contrast, PPD-B induced a DTH response (of ≥4 mm) in 10/15 (0.67; 95% CI: 0.42, 0.85) of BCG vaccinates and 2/15 (0.13; 95% CI: 0.03, 039) unvaccinated controls that would be considered as reactors per the traditional single intradermal test (Figure 3). However, due to the equally high skin responses to PPD-A observed in BCG vaccinated calves, none of the vaccinated animals were classified as reactors under standard interpretation conditions of the comparative cervical intradermal test (PPD-B minus PPD-A > 4 mm) (Figure 3). Of note, the animals in the unvaccinated control group that exhibited ≥ 4 mm PPD-B stimulated skin induration responses also had a high (≥4 mm) PPD-A response (Figure 3).

## Discussion

BCG is the most widely used of all vaccines today (and the only vaccine available against human TB). While BCG is licensed for use in wildlife (23), it is not licensed for use in domestic livestock because vaccination results in sensitization to the PPD-based skin and IGRA tests. Hence, the development of a diagnostic test that overcomes these limitations of the OIE-recommended tuberculin-based skin tests to differentiate infected from vaccinated animals is considered an essential prerequisite for the implementation of BCG vaccination of cattle, particularly in settings where traditional test and slaughter remain unfeasible (24).

In the early 1900s, the parent of BCG *M. bovis* isolate was made to undergo ~230 serial passages that resulted in its loss of virulence in various animal models and consideration for use as a vaccine in both cattle and humans (9, 25). Subsequent genomic analyses revealed that the primary attenuating mutation was likely the loss of an approximately 9.5-kb locus [termed as “Region of Difference 1” (RD1)] prior to the clinical use of BCG in 1921. All modern BCG vaccine substrains lack RD1, and the complementation of RD1 has been shown to result in a protein expression profile that is similar to that of the virulent strain, confirming that this region harbors some of the major antigenic targets identified thus far, including ESAT-6 and CFP-10 (26). RD1 also encodes the esx1 secretion system, therefore resulting in loss of immunogenicity of proteins dependent on this secretion system, e.g., Rv3615c, in BCG. Since these antigens are present in the genome of *M. bovis* but absent or not immunogenic in BCG, they represent promising candidates for the development of a DIVA diagnostic test for bTB, as has been previously shown (14, 19).

We recently developed a synthetic peptide-based DST (representing ESAT-6, CFP-10 and Rv3615c) and demonstrated its considerable sensitivity in reliably detecting *M. bovis*-infected animals in skin tests and in *in vitro* assays, while also maintaining high specificity in naïve controls (16). However, the specificity of DST in BCG-vaccinated animals in skin tests, in bTB endemic settings, was unknown. The results of the current investigation considerably extend the prior observation of specificity of DST in BCG-vaccinated cattle in IGRA by now establishing the specificity of the DST as a skin test reagent in BCG-vaccinated and vaccine naïve calves, which is essential if DST were to be used as a DIVA diagnostic in field settings (16).

The IGRA results from these 3- to 6-month-old cross-bred calves (Jersey X. Zebu) in India to PPD-A and PPD-B at the start of the study (0th week, pre-vaccination) suggest pre-sensitization from high levels of exposure to environmental mycobacteria and pathogenic mycobacteria, including *Mycobacterium avium* ssp. *paratuberculosis* in these geographies (27, 28). While in general, due to non-specific IFN-γ production, minimum eligible age for BOVIGAM^TM^ assays is 6 months, the whole blood cells derived from pre-BCG vaccinated calves remained unreactive to DST (Table 1 and Figure 2) (29). Also, the equivalent PPD-B and PPD-A skin responses observed in BCG-vaccinated calves in our studies was noteworthy since PPD-B, which is derived from a *M. bovis* strain, might be expected to share greater antigenic similarity to BCG than a *M. avium* subsp. *avium* strain from which PPD-A is derived (11). Contrary to this expectation, the results of our studies suggest that while BCG likely stimulates a broadly cross-reactive (non-specific) response to mycobacteria, the specificity is driven by antigens, such as those that are encoded by or dependent on the RD1 locus that is missing in BCG. In this context, since the DST consists of antigens present only in members of the *M. tuberculosis* complex and a few other pathogenic mycobacteria (*M. leprae* and *M. kansasii*), it would be expected to provide superior specificity over PPD-B alone as observed in our current studies. It is also important to note that given the cross-reactivity between PPD-B and PPD-A, there are serious implications for the sensitivity of CCT in settings such as in India where there is likely a high burden of environmental mycobacteria. In such conditions of high background environmental mycobacterial exposure that drives PPD-A reactivity, CCT would result in a high proportion of false-negative results whereas SIT, performed with PPD-B alone, would result in a high proportion of false positives (12). Hence, although the current study suggests that BCG-vaccinated calves would be considered to be non-responders in the CCT assay, the general utility of CCT in conditions such as those encountered in India and other countries with high environmental burden remains unclear.

Another interesting observation is that animal IDs 69 and 80 remained relatively unresponsive to BCG immunization based on the trajectories of their immune responses as measured by IGRA over time (Supplementary Table 1). While the reason for this is unclear, it is crucial for future studies to identify biomarkers to confirm vaccine-induced protection in order to formulate evidence-based approaches to the development of a bTB control program. Importantly, in contrast to PPDs, DST was highly specific—revealing no cross-reactivity with environmental mycobacteria in both whole blood IGRA and in the skin test. The observation that DST specificities are not impaired under field conditions in highly endemic settings is encouraging and suggests that the DST may overcome the specificity limitations of traditional PPD-based skin tests both in the face of both BCG vaccination and due to sensitization with saprophytic mycobacteria that also likely complicate interpretation and clinical sensitivity of the CCT. Although the group sizes of the current study are relatively low, these are crucial proof-of-concept experiments that demonstrate the much-needed DIVA capability of a diagnostic test in order to enable the implementation of livestock vaccination programs in regions in which bTB is endemic and remains uncontrolled, and where test and slaughter remain unfeasible. We note that it is crucial to evaluate the performance of DST in vaccinated animals that subsequently become infected.

In summary, this study confirms that DST allows not only discrimination between infected animals and those environmentally sensitized but also its utility as a much-needed DIVA test reagent in a challenging geographical context. Thereby, the DST has the potential to overcome a major hurdle for the implementation of BCG cattle vaccination programs as an intervention strategy for disease control.

## Data Availability Statement

All datasets generated for this study are included in the article/Supplementary Material.

## Ethics Statement

The animal study was reviewed and approved by Institutional Animal Ethics Committee (IAEC) at TANUVAS and Committee for the Purpose of Control and Supervision of Experimental Animals (CPCSEA; F. No. 25/31/2017-CPCSEA).

## Author Contributions

SSr, DG, KK, MVe, DB, MVo, and VK conceptualized and designed the study. SSr, SSu, SSh, MJ, and SK performed the experiments. KR, VM, SSe, and MVe carried out the field work. SSr analyzed the data. SSr drafted the paper. SSr, DB, MVo, and VK contributed to writing. All authors reviewed the manuscript.

## Conflict of Interest

Animal and Plant Health Agency (APHA), the employer for MVo, holds three patents (patent numbers WO/2009/060184, WO/2011/135369, and WO/2012/010875) for the use of Rv3615c in diagnostic tests for bTB. In addition, APHA (MVo) and Penn State (SSr and VK) are in the process of intellectual property protection filings for the DST. The remaining authors declare that the research was conducted in the absence of any commercial or financial relationships that could be construed as a potential conflict of interest.
